# Comparison of a combination test (1 μg ACTH test plus glucagon test) versus 1 μg ACTH test and glucagon test in the evaluation of the hypothalamic-pituitary-adrenal axis in patients with pituitary disorders

**DOI:** 10.20945/2359-3997000000266

**Published:** 2020-06-19

**Authors:** Kursad Unluhizarci, Emel Oguz Kokoglu, Ayşa Hacioglu, Zuleyha Karaca, Fahrettin Kelestimur

**Affiliations:** 1 Department of Endocrinology Erciyes University Medical School Kayseri Turkey Department of Endocrinology, Erciyes University Medical School, Kayseri, Turkey; 2 Department of Endocrinology Yeditepe University Medical School Istanbul Turkey Department of Endocrinology, Yeditepe University Medical School, Istanbul, Turkey

**Keywords:** Low-dose ACTH test, glucagon test, pituitary, adrenal insufficiency

## Abstract

**Objective:**

To investigate whether a combination of the low-dose (1 µg) adrenocorticotropin (ACTH) stimulation test and glucagon stimulation test (GST) could overcome the problem of equivocal results with the GST or ACTH test alone in patients with pituitary disorders.

**Subjects and methods:**

The study included 41 adult patients with pituitary disorders and 20 healthy subjects who underwent evaluation of cortisol response to ACTH, GST, and a combination of both tests. Blood samples for cortisol measurement were obtained at baseline and 30, 60, 90, and 120 minutes after intravenous administration of ACTH 1 μg and 90, 120, 150, 180, 210, and 240 minutes after subcutaneous injection of glucagon 1 mg. The combination test was performed by injecting ACTH 1 µg at the 180-minute time point of the GST, with blood samples for cortisol measurement obtained at 210 and 240 minutes.

**Results:**

Overall, 28 patients with normal cortisol response to both tests also had a normal cortisol response to the combination test. Ten patients with adrenal insufficiency in both tests also had adrenal insufficiency in the combination test, including a patient who had a peak cortisol value of 12.4 µg/dL (which is the cutoff value for the combination test). Two patients with adrenal insufficiency in the ACTH stimulation test and one patient with adrenal insufficiency in the GST had normal cortisol responses to the combination test.

**Conclusion:**

By using an appropriate cutoff value, the combination test may offer additional information in patients with equivocal results in the GST and ACTH stimulation test.

## INTRODUCTION

The hypothalamic-pituitary-adrenal (HPA) axis should be evaluated appropriately in patients with pituitary disorders since the decision for glucocorticoid replacement therapy is based on the results of some tests, including the adrenocorticotropin (ACTH) stimulation test, insulin tolerance test (ITT), or glucagon stimulation test (GST) ( 1 , 2 ). Although basal serum cortisol levels may be helpful in this regard, dynamic tests of the HPA axis are usually required in patients with suspected HPA axis insufficiency. The ITT is considered the gold-standard test to evaluate the HPA axis in patients with pituitary disorders but is a difficult test and requires medical supervision. Moreover, this test is contraindicated in patients with epilepsy and those with cerebrovascular or cardiovascular disorders, and must be cautiously performed in elderly patients. The ACTH stimulation test and the GST are some alternatives to the ITT. The low-dose (1 μg) ACTH stimulation test has been suggested as a sensitive and reliable alternative to the ITT ( 3 - 6 ). On the other hand, the GST has been used more frequently in the evaluation of the growth hormone (GH) axis due to the unavailability of GH-releasing hormone. The GST offers an opportunity to assess both the HPA and GH axes, making it an attractive alternative ( 7 ). However, the proper establishment of cutoff levels for cortisol is needed for correct interpretation of GST results ( 8 - 10 ).

In the present study, the first of its kind, we compared the combination of low-dose ACTH and GST (“combination test”) versus each test alone in the evaluation of the HPA axis in patients with pituitary disorders. The aim of the study was to determine whether the combination test could overcome the problems of equivocal results with the GST and ACTH test.

## SUBJECTS AND METHODS

The study was approved by the Ethics Committee and the Institutional Review Board of the Erciyes University Medical School, and informed consent was obtained from each patient.

In all, 41 adult patients (26 women, 15 men) with pituitary disorders (non-functional adenoma, prolactinoma, Sheehan’s syndrome etc.) and 20 healthy subjects (7 men, 13 women) were included in the study. Patients with diabetes mellitus or a diagnosis of Cushing’s syndrome were not included.

The low-dose ACTH, GST, and combination tests were performed on separate days (with at least a 48-hour interval) after overnight fasting. All patients were euthyroid when the dynamic tests were performed. Glucocorticoid replacement therapy was withdrawn under close supervision for at least 24 hours before the tests. Blood samples for measurement of cortisol were obtained at baseline and at 30, 60, 90, and 120 minutes after intravenous administration of ACTH 1 μg. In the low-dose ACTH test, the lowest peak cortisol value in control subjects (14.6 μg/dL) was considered as the cutoff value for adrenal insufficiency.

The GST was performed with subcutaneous injection of glucagon 1 mg (Novo Nordisk, Bagsvaerd, Denmark). Blood samples for measurement of cortisol were obtained at 90, 120, 150, 180, 210, and 240 minutes after glucagon injection. The lowest peak cortisol value in control subjects (9.7 μg/dL) was considered as the cutoff value for adrenal insufficiency.

The combination test was performed with intravenous injection of ACTH 1 μg at the 180-minute time point of the GST, and blood samples for cortisol measurement were obtained at 210 and 240 minutes. Receiver operating characteristics (ROC) analysis was performed to determine the optimal cutoff value for the diagnosis of adrenal insufficiency, and a cortisol value of 12.4 mg/dL was obtained with 83% sensitivity and 100% specificity. In contrast, the lowest cortisol response in the combination test among healthy subjects was 15.7 μg/dL.

### Assay

Serum levels of cortisol, prolactin, FSH, LH, estradiol, testosterone, free T3, free T4, and TSH were measured using the electrochemiluminescence immunoassay (ECLIA) method with a commercially available kit (Cobas, Roche Diagnostics, Mannheim, Germany), and serum GH, IGF-1, and ACTH levels were measured using two-site chemiluminescent immunometric assay with a commercially available kit (Immulite 2000, Siemens Healthcare Diagnostics, Gwynedd, UK). The intra- and interassay coefficients of variation were, respectively, 1.7% and 2.2% for cortisol, 0.8% and 1.8% for prolactin, 2.6% and 3.6% for FSH, 1.2% and 2.0% for LH, 6.1% and 7.0% for estradiol, 4.1% and 4.4% for testosterone, 2.0% and 2.5% for free T3, 1.4% and 1.8% for free T4, 1.1% and 3.0% for TSH, 3.5% and 4.6% for GH, 3.0% and 3.9% for IGF-1, and 6.7% and 8.2% for ACTH.

### Statistical analysis

The statistical analysis was performed with the software IBM SPSS, version 15 (IBM Inc., Chicago, IL, USA) program. Normally distributed data are presented as mean ± standard error of the mean (SEM) and non-normally distributed variables as median (25%-75%). Paired and unpaired Student’s *t* tests were used to compare parametric variables, while the Mann-Whitney U and Wilcoxon tests were used for non-parametric variables. ROC curve analysis was conducted to determine a cutoff level for peak cortisol response in the combination test. In the ROC analysis, we excluded patients with discordant results in the GST and ACTH test. P values < 0.05 were considered statistically significant.

## RESULTS

Both patient and control groups were comparable in terms of age, gender, waist circumference, and body mass index ( Table 1 ). The most common causes of pituitary disease were non-functional pituitary adenoma and empty sella syndrome. Baseline free T3, free T4, cortisol, FSH, and LH values were lower in the patient compared with the control group ( Table 2 ). In the patient group, 8, 19, and 7 patients were receiving glucocorticoid, thyroid, and gonadal hormone replacement therapies, respectively. Two patients with adrenal insufficiency were not on glucocorticoid therapy because of lack of compliance. Glucocorticoid replacement therapy was withdrawn under close supervision for 24 hours before the tests.

**Table 1:** Clinical characteristics of the patient and control groups

	Patient group (n = 41)	Control group (n = 20)	P value
Age (years)	46.8 ± 2.0	46.5 ± 2.2	NS
BMI (kg/m^2^)	30.8 ± 0.9	30.2 ± 0.9	NS
Waist (cm)	106.1 ± 2.3	101.8 ± 2.7	NS
Men/Women	15/26	7/13	NA

NS: not significant; NA: not applicable. Data are shown given as mean ± standard error of the mean (SEM).

**Table 2:** Baseline hormone levels in the patient and control groups

Hormones	Normal values	Patient group (n = 41)	Control group (n = 20)	P value
FT3 (pg/mL)	2-4.4-	2.8 ± 0.8	3.3 ± 0.1	< 0.05
FT4 (ng/dL)	0.93-1.97	1.05 (0.9-1.1)	1.23 (1.08-1.4)	< 0.05
TSH (μIU/mL)	0.27-4.2	1.24 (0.2-2.2)	1.66 (1.07-2.3)	NS
GH (ng/mL)	0-1	0.05 (0.05-0.2)	0.06 (0.05-0.14)	NS
ACTH (pg/mL)	0-46	17.7 (13.5-27)	15.3 (11.5-24.0)	NS
Cortisol (μg/dL)	6.2-18	6.5 (2.4-7.5)	10.2 (7.6-14.7)	< 0.05
Prolactin (ng/mL)	4.8-23	13.8 (9.9-26.5)	10.9 (8.6-13.9)	NS
FSH (mIU/mL)		4.3 (1.0-9.1)	9.7 (4.3-70.8)	< 0.05
LH (mIU/mL)		3.4 (0.4-7.4)	6.7 (4.3-34.2)	< 0.05
IGF-1 (ng/mL)	109-284	109.3 ± 11.1	106.9 ± 5.8	NS

NS: not significant. Values are presented as mean ± standard error of the mean (SEM) or median (25%-75%) where appropriate

Overall, 28 patients with normal cortisol response to both (ACTH and GST) tests also had normal cortisol response to the combination test. Ten patients with adrenal insufficiency in both tests also had adrenal insufficiency in the combination test, including a patient who had a peak cortisol value of 12.4 μg/dL (the cutoff value for the combination test) ( Table 3 ). Two patients with adrenal insufficiency (peak cortisol responses of 13.4 and 13.1 μg/dL) in the ACTH stimulation test and one patient with adrenal insufficiency in the GST (peak cortisol response of 8.7 μg/dL) had normal cortisol responses in the combination test ( Table 4 ). These patients were not truly adrenal insufficient. The peak cortisol responses in the combination test were slightly higher than those in the ACTH test in patients either with or without adrenal insufficiency ( Table 5 ).

**Table 3:** Peak cortisol responses (in μg/dL) to ACTH, glucagon stimulation test (GST), and combination test in 10 patients with adrenal insufficiency

Patients	ACTH stimulation test	GST	Combination test
1	9.6	7.1	12.4
2	6.2	2.8	4.5
3	4.1	5.9	9
4	2.2	1.7	2.9
5	3.5	1.6	3.3
6	3	4.5	4.6
7	0.3	0.2	0.7
8	3.9	1.7	4
9	1.2	0.7	1.1
10	4.6	3.6	5.9

**Table 4:** Data of 3 patients with discordant results in the GST and ACTH stimulation test

	ACTH stimulation test	GST	Combination test
Patients with AI in the ACTH test alone	13.4	11.7	19.3
	13.1	27	17.1
Patients with AI in the GST alone	16	8.7	16.8

AI: adrenal insufficiency; GST: glucagon stimulation test. Values are presented in μg/dL.

**Table 5:** Peak cortisol responses (in μg/dL) in stimulation tests in the patient group

	Adrenal insufficiency (n = 10)	Adrenal sufficiency (n = 28)	P value
ACTH stimulation test	3.7 (2.0-5.0)	18.0 (16.7-21.6)	< 0.05
GST	2.3 (1.4-4.8)	14.2 (11.7-18.7)	< 0.05
Combination test	4.2 (2.4-6.7)	20.4 (18.1-22.8)	< 0.05

GST: glucagon stimulation test. Values are presented as median (25%-75%).

## DISCUSSION

The diagnosis of adrenal insufficiency has paramount importance in patients with pituitary disorders since this condition results in decreased quality of life and increased risk of adrenal crisis and mortality in stressful conditions. However, the diagnosis may be delayed for several years due to nonspecific signs and symptoms. In a cross-sectional study in 216 patients with both primary and secondary adrenal insufficiency, symptoms before diagnosis were present for more than 1 year in 47% and more than 5 years in 20% ( 11 ). Upon suspicion of adrenal insufficiency, biochemical testing is required to confirm the diagnosis. The initial step in the evaluation is the measurement of baseline morning serum cortisol level. Baseline cortisol measurement may not diagnose adrenal insufficiency in some patients, and a few dynamic tests may be required. However, some controversial issues remain in the assessment of ACTH insufficiency with dynamic stimulation tests in patients with pituitary disorders, although the final diagnosis usually relies on these tests ( 12 - 14 ).

The low-dose ACTH stimulation test correlates well with the ITT ( 5 , 15 ), which is considered the gold-standard test. Recently, Ospina and cols. ( 16 ) conducted a meta-analysis to test the diagnostic accuracy of the standard-dose and low-dose ACTH stimulation tests in patients with either primary or secondary adrenal insufficiency. The authors suggested that in patients with pituitary disorders, both tests are more helpful in ruling in the condition when positive, but are not as reliable in ruling out the condition when negative. The authors also suggested that, although ACTH stimulation tests are helpful, they are not perfect and can be misleading in some cases.

On the other hand, GST is a reliable alternative test to assess cortisol secretion. Since the way and ability of each test to stimulate cortisol secretion are clearly different, the same cutoff levels for cortisol cannot be used to evaluate the HPA axis ( 17 ). An advantage of the GST is its ability to evaluate both GH and ACTH release. We have previously shown that the cortisol cutoff value in healthy adults undergoing the GST should be 9.1 μg/dL ( 2 ). In that study, the lowest peak cortisol responses obtained after low-dose ACTH and GST were 12.5 μg/dL and 9.1 μg/dL, respectively, in volunteers with all cortisol responses greater than 20 μg/dL after standard-dose ACTH stimulation test. This result indicates that the GST and the low-dose ACTH test may be used with appropriate cutoff values to evaluate the HPA axis. A similar cutoff value for cortisol (10.0 μg/dL) in the GST has been suggested by Berg and cols. ( 8 ), with > 95% specificity and 72% sensitivity for adrenal insufficiency in patients after pituitary surgery. A recent study by Hamrahian and cols. ( 18 ) also considered the GST to be a reliable test to evaluate secondary adrenal insufficiency when appropriate cortisol cutoff values are used (9 μg/dL for fixed-dose GST and 11 μg/dL for weight-based dosing GST). In the present study, we found a cutoff value for cortisol of 9.7 μg/dL, which is quite similar to the previously suggested values. In some patients, the HPA axis may not be evaluated reliably by a single test, and more than one test may be required to diagnose adrenal insufficiency, as shown recently ( 16 ). Thus, we aimed in the present study to verify whether a combination of GST and ACTH stimulation test would provide multi-targeted stimuli and, more importantly, verify if the combination test could overcome the equivocal results obtained with each test alone. Theoretically, the combination of glucagon and ACTH is expected to lead to a stronger stimulation of the adrenal axis. Accordingly, our data showed that among healthy adults, the lowest cortisol value in the combination test is higher than that in the ACTH test. However, we preferred to find a cutoff value for the combination test by using ROC analysis. No data regarding the value of the combination test is available in the literature, so we could obtain no data to use as a cut-off value for this test. ROC analysis is used to provide/establish the maximum specificity and sensitivity for a given test and compare the success of two or more tests for diagnosis. Because of these reasons, we opted to perform a ROC analysis, which showed 83% sensitivity and 100% specificity for a cortisol value of 12.4 μg/dL.

In all, 10 patients had adrenal insufficiency in both tests. These patients also had adrenal insufficiency in the combination test (one patient had a peak cortisol response at the cutoff level), suggesting the combination test to be a useful and reliable tool to discriminate patients with adrenal insufficiency. Moreover, the combination test showed adequate cortisol response in 28 patients who had adequate cortisol responses to both tests. In only 3 patients (7.3%), both tests yielded contradictory results, although the patients were not clinically adrenal insufficient. In these patients, the combination test resulted in an adequate cortisol response. We can speculate that in patients without an apparent diagnosis of adrenal insufficiency, the combination test can safely identify the diagnosis.

All tests in this study were performed in the same patients and control subjects under identical conditions. Therefore, the differences in cortisol responses cannot be attributed to individual differences in the methods used for cortisol measurement. The highest cortisol response was seen in the combination test. This suggests that a direct stimulatory effect of ACTH on the adrenal glands may be seen in patients with an HPA axis already stimulated by another mechanism (glucagon stimulation in these cases). Another remarkable point of this study was that, similar to our previous results ( 17 ), we confirmed that the cutoff values for cortisol should not be used universally for all tests, and that cutoff levels for HPA axis insufficiency should be individualized for each test.

In summary, our results suggest that patients with clinically apparent secondary adrenal insufficiency may not require any tests, while GST/low-dose ACTH test may be used to confirm the diagnosis. However, by adopting an appropriate cutoff value, the combination test may offer additional information in patients with equivocal results in the GST and ACTH stimulation test. Moreover, by performing the combination test, we can save a day of the patient’s time and evaluate concomitantly the GH axis instead of performing GH stimulation tests.
